# Supplementary material for: Shared Decision Making with Acutely Hospitalized, Older Poly-Medicated Patients: A Mixed-Methods Study in an Emergency Department
Source: Int J Environ Res Public Health. 2022 May 25;19(11):6429. doi: 10.3390/ijerph19116429 (PMC9180707; doi:10.3390/ijerph19116429)
Supplement: Supplementary file 1 [file ijerph-19-06429-s001.zip › ijerph-1730961-supplementary.pdf]

Table S1. Checklist of Mixed Methods Elements in a Submission to Advance the Methodology of Mixed Methods Research

**Manuscript title**<br>" Shared Decision Making with Acutely Hospitalized Older Poly-medicated Patients: A Mixed- method Study in an Emergency Department"			Yes	No	Page number (if not applicable, indicate NA)
**Title**					
	1	Does the title directly indicate or sufficiently allude to the methodological contribution of the article?	X		Front page
**Abstract**					
	2	Does the abstract include an explicit statement about a methodological challenge or issue in the field that will be addressed in the article?	X		Front page
	3	Does the abstract indicate the methodological/theoretical contribution of the article to the field of mixed methods research?		X	
**Main text of the article**					
	4	Does the article have a clear writing style with sufficient headers and sub- headers such that the reader can readily follow the flow and argumentation?	X		NA
	5	Does the text in the background reiterate and expand upon the methodological challenge or issue as identified in the abstract?	X		p.2, 3-top
	6	Does the background contain a rigorous review and citations of relevant and recent mixed methods literature to support examining the methodological aim?		X	
	7	Does the background include an explicit methodological aim?	X		See 2.2. study design p. 3
	8	Does the background contain an explication of the article's structure and methodological points that will be addressed?	X		See 2.2. study design p. 3
	9	In the body of article, are each of the methodological points identified in #8 addressed persuasively in the order specified?	X		
	10	Does the article include a strategy to convey the overall complexity of the topic or study phenomenon such as a figure or illustration?	X		Figure 1 page 3 + Table 5 (Meta-inferences between datasets) p.9
	11	In the discussion, are the explicit points made in #8 synthesized together to logically support the overarching methodological aim?	X		p. 14, 15
	12	Does the discussion section include a specific subsection "Contribution to the Field of Mixed Methods Research" that reviews the points made and extant literature to articulate the articles novel contribution(s) to mixed methods?		X	
	13	Does the article have a discussion of the methodological limitations?	X		Page 15 Methods strengths and limitations
	14	Does the discussion section include recommendations for future mixed methods inquiry based on the paper's unique contribution or limitations?		X	
	15	Have the references been cited according to the current American Psychological Association style?		X	NA
**Additional elements for empirical methodological articles only**					
	16	Does the background of the article include explicit statements of both the methodological aim and purpose of the empirical study separately?	X		In objectives p. 3

	17	Does the description of the methods include sufficient detail about the procedures used and present these in a logical order?	X		In study design p. 3 + p.4-7
	18	Does the submission include a procedural diagram of the data collection and analysis procedures as a figure?	X		Figure 1 p. 3
	19	Does the submission include a table, matrix or visual structure, e.g., joint display, to illustrate integration and interpretation of the qualitative and quantitative findings?	X		Table 5 p. 9
	20	Does the discussion articulate how the use of a mixed methods approach advanced a greater understanding of the substantive topic compared to using a monomethod approach?	X		Page 15 Methods strengths and limitations

Note. Adapted from Fetters and Freshwater (2015a) and Fetters and Molina-Azorin (2019).

Table S2. Themes and sub-themes from the patient interview

Theme	Sub-themes
Sparse communication about medicine in the acute phase	Things were moving a little too quickly<br>Difficult to recall information in the acute phase<br>No need to discuss medication because everything is written down
Talk about side-effects	Crucial to discuss side-effects<br>No one enquired about side-effects<br>Active patients want to investigate different alternatives
Preferences for deprescribing	Most patients prefer to take less medicine<br>Difference in the active and passive patients' behavior
Power disparities prevents dialogue about medicine	Inquiring is to show vulnerability
Patients lack knowledge of their medicine	Some patients prefer more knowledge about the effect of their new medicine<br>Active patients are skeptical of new medicine, and wants more information why their medication was changed<br>Active patients seeking further information concerning or with respect to their medicine on the internet after discharge

Table S3. Analysis example from transcript to theme

Interview transcription	Code	Sub-theme	Theme
Patient: *"No, no one asked about medicine. They have everything in writing, so there is nothing to discuss. They simply glance at their screens to know what you get and don't get"* (Pt ID # 2).	No one asked about the patient's medicine in the ED.	No need to discuss medi-cation because everything is written down.	Sparse com-munication about medicine in the acute phase of an hospitalization
